# Comparison between the world health organization (WHO) and international society of hypertension (ISH) guidelines for hypertension

**DOI:** 10.1080/07853890.2022.2044510

**Published:** 2022-03-15

**Authors:** Pringgodigdo Nugroho, Hubert Andrew, Kelvin Kohar, Chairina Azkya Noor, Aida Lydia Sutranto

**Affiliations:** a Division of Nephrology and Hypertension, Department of Internal Medicine, Dr Cipto Mangunkusumo Hospital, Jakarta, Indonesia; b Faculty of Medicine, Universitas Indonesia, Jakarta, Indonesia

**Keywords:** Hypertension, guidelines, world health organisation, international society of hypertension, comparison, low- and middle-income countries, high-income countries

## Abstract

The global burden of hypertension remains an unsolved problem, especially in low- and middle-income countries (LMICs). For this reason, clinical practice guidelines containing the latest evidence-based recommendations are crucial in the management of hypertension. It is noteworthy that guidelines simply translated from those of high-income countries (HICs) are not the solution to the problem of hypertension in LMICs. Among the numerous guidelines available, those of the World Health Organisation and the International Society of Hypertension are the latest to be published as of the writing of this article. In this review, we conducted both general and specific comparisons between the recommendations supplied by both guidelines. Differences in aspects of hypertension management such as the timing of antihypertensive initiation, assessment of comorbidities and cardiovascular risk factors, pharmacological therapy selection, and blood pressure target and reassessment are explored. Lastly, the implications of the differences found between the two guidelines in both LMICs and HICs are discussed.

**Key messages**
- Currently, with low treatment and control rates, hypertension remains a burden in low- and middle-income countries (LMICs).
- The lack of customised guidelines for LMICs cannot be solved simply by adopting guidelines from high-income countries.
- The World Health Organisation (WHO) recently published a clinical guideline for the pharmacological management of hypertension in LMICs. We compare select recommendations from the guidelines to those published by the International Society of Hypertension.

## 1. Introduction

The 2021 World Health Organisation (WHO) “Guidelines for the pharmacological treatment of hypertension in adults” and “The 2020 International Society of Hypertension (ISH) Global Hypertension Practice Guidelines” are the two recently updated recommendations on clinical approaches for the management of hypertension [1,2]. Both guidelines are considered to provide up-to-date evidence-based sources of information from recently published studies. However, the ISH does not specify the methodology used in formulating their guidelines. On the contrary, the WHO has a dedicated section elaborating on their methodology in developing the guidelines. Nevertheless, both guidelines are critically reviewed by an external panel of experts from various backgrounds.

Clinical practice guidelines consist of all recommendations regarding the diagnosis and treatment of a medical condition. They are systematically arranged to ensure that doctors treat patients as per appropriate standards of treatment and care. A good practice guideline should be updated based on current knowledge and developed systematically using reliable methodology. It should be used as a justification, not as an obligation while treating patients [3].

The WHO, in liaison with the ISH, began the development of guidelines for hypertension management in 1999 [4]. In 2003, the organisations released new collaborative statements on their previous guidelines, as more studies on hypertension became widely available [5]. This was their last joint guideline on the management of hypertension. Recently, the WHO and the ISH published separate hypertension guidelines in 2020 and 2021, respectively. However, the reason for this separation remains unknown.

Hypertension is the leading cause of cardiovascular (CV) disease (CVD) and premature death worldwide, especially in low- and middle-income countries (LMICs) [6]. According to a meta-analysis by the Non-Communicable Diseases (NCDs) Risk Factor Collaboration, the global prevalence of hypertension has doubled from 1990 to 2019. The control and treatment rates among people are still low, particularly in LMICs^7^ .Therefore, updated clinical guidelines for hypertension management are still in demand. Another major issue in this clinical field is the variety of available guidelines, which could potentially lead to confusion in choosing the most suitable one. Herein, we compare the guidelines put forth by the WHO and the ISH, and evaluate their advantages and shortcomings.

## 2. Discussion

We primarily compared two hypertension guidelines, the WHO’s “Guidelines for the pharmacological treatment of hypertension in adults” and the ISH’s “The 2020 International Society of Hypertension (ISH) Global Hypertension Practice Guidelines” and conducted a broad literature search through the PubMed database for additional context. Additionally, we expanded the scope of the inquiry by manually reviewing related references.

### 2.1. Comparison between the guidelines

#### 2.1.1. Defining the disease

According to the ISH, blood pressure (BP) is classified into four categories: normal (<130/85 mmHg), high-normal (130–139/85–89 mmHg), grade 1 hypertension (140–159/90–99 mmHg), and grade 2 hypertension (≥160/100 mmHg) [2]. This classification is not included in the WHO guidelines, which focus more on pharmacological treatment [1]. Thus, it is important to precisely define hypertension. The ISH designated an office BP reading of more than 140/90 mmHg as hypertensive [2]. By contrast, the latest WHO guidelines do not contain this fundamental definition, although the level of BP requiring treatment has been specified. The guideline only states that hypertension can be defined by systolic and diastolic BP or the reported use of antihypertensives. While evidently lacking, the WHO guideline explicitly mentions that it does not address BP measurement and the diagnosis of hypertension, among other issues. Instead, it mainly focuses on pharmacological treatment of hypertension.

#### 2.1.2. Starting antihypertensives

The other major issue in the treatment of hypertension is when to start therapy. The initiation of pharmacological treatment is usually considered after lifestyle interventions prove ineffective in the management of high BP [8]. A recent analysis covering 99% of the global population revealed that more than half of those with hypertension are not receiving the treatment they need, although it is widely regarded as effective and inexpensive. Furthermore, a considerable disparity in treatment coverage exists among countries. More than 70% of patients in high-income countries (HICs), such as South Korea and Canada receive treatment, in contrast to less than a quarter of patients in LMICs, such as Nepal and Indonesia [7].

Regarding the initiation of antihypertensives, the WHO sets a lower threshold in comparison to the ISH for those with and without high CV risk (Table 1). For those without CV risk, the WHO recommends starting antihypertensives for patients with grade 1 hypertension (≥140/90 mmHg). By contrast, the ISH endorses lifestyle interventions for such patients, reserving pharmacological treatment for high-risk conditions only, such as coronary artery disease [1,2].

**Table 1.** Fundamental differences in treatment and diagnostic methods.

Differences in the guidelines						
Parameters	World Health Organisation (WHO)			International Society for Hypertension (ISH)		
Office blood pressure (BP) defined as hypertensive	Systolic (mmHg)	and/or	Diastolic (mmHg)	Systolic (mmHg)	and/or	Diastolic (mmHg)
	Not mentioned			≥140		≥90
BP threshold for pharmacological treatment (General population)	Confirmed diagnosis of hypertension			Grade 2 hypertension*		
	≥140		≥90	≥160		≥100
BP threshold for pharmacological treatment (High-risk population)**	130–139		any	Grade 1 hypertension*		
				140–159		90–99
Comorbidity and secondary HT screening	Blood test, urine dipstick, and ECG are recommended if initiation of any pharmacological therapy is not postponed (especially in low-resource area)			CV medical and family history, CV physical examination, blood and urine tests, 12-lead ECG, and various imaging and functional tests		
CV risk assessment	Only for patients with high-normal blood pressure (SBP 130–139)			Every hypertensive patient		
Therapy follow-up	Monthly follow-up after initiation until controlled, followed by once every 3–6 months			Not mentioned		
**Similarities between the guidelines**						
	Recommends single-pill combination as initial therapy					
	Does not address specific non-pharmacological interventions, but considers them as a part of a comprehensive treatment			Emphasizes and provides a specific target for each modifiable risk factor		

BP: blood pressure; CV: Cardiovascular; ECG: electrocardiogram; ISH: International Society for Hypertension; HT: hypertension; SBP: systolic blood pressure; WHO: World Health Organisation.

*Based on the ISH grading of blood pressure.

**High-risk population as defined by the ISH (cardiovascular diseases, diabetes mellitus, chronic kidney disease, etc.).

Care must be taken to not overtreat such patients, as more harm than benefit is observed with intensive treatment of those with lower CV risk. Philip *et al.*, using data from the SPRINT trial, showed that the harm from significant adverse effects experienced by patients with a calculated CV risk of ≤ 18.1% in intensive treatment is still greater than the benefit received from the reduction of primary outcome events (myocardial infraction, stroke, death from CV causes, among others) [9]. Among patients at intermediate CV risk, medication also does not seem to significantly lower the rate of CV events. In a study with over 12,000 patients, Lonn *et al.* demonstrated that candesartan plus hydrochlorothiazide therapy did not lower the risk of major CV events when compared with placebo in patients without CVD at intermediate CV risk [10].

On the contrary, a recent meta-analysis authored by The Blood Pressure Lowering Treatment Trialists’ Collaboration showed that a 5-mmHg reduction of SBP decreased the risk of major CV events by 10%. Interestingly, the risk reduction was present regardless of CV risk, even in patients with BP levels currently not considered for treatment [11]. Nevertheless, both guidelines concur on withholding BP-lowering drugs in patients with high-normal BP readings and low-moderate CV risk and on prioritising lifestyle modifications in such individuals. Indeed, conclusions from the previously mentioned meta-analysis also emphasised the importance of reducing CV risk rather than the BP itself.

Meanwhile, for those with high CV risk, the WHO states that a systolic BP (SBP) of 130–139 mmHg is an indication to introduce antihypertensives, whereas the ISH recommends grade 1 hypertension (140/90–159/99 mmHg) as an indication. Although both guidelines consider CV risk before the initiation of BP-lowering drugs, the benefits of antihypertensives are minimal at best, even in patients with high CV risk, if the baseline BP is below 140/90 mmHg. Hence, a patient’s BP must be considered when deciding the timing of treatment initiation [12].

Besides the patient’s BP and CV risk, another factor to consider before starting treatment is the cost burden, which is especially relevant in LMICs. It may not be financially sustainable or cost-effective for health systems with limited resources to fully follow treatment guidelines for low-risk patients, which initiates antihypertensives at an earlier stage. A cost-effectiveness analysis of the threshold of initiation of pharmacological treatment, such as the ones performed by Constanti [13] *et al.* on the 2019 National Institute for Health and Care Excellence hypertension guideline and Gaziano [14] on the 2001 South African Hypertension Society hypertension guideline, may be an insightful asset.

Overall, the benefits, risk reduction, need for lifelong medication, and side effects of antihypertensives along with individual comorbid conditions should be evaluated before starting antihypertensives.

#### 2.1.3. Out-of-Office BP measurements

Out-of-office BP measurements, that is, ambulatory BP measurements (ABPM) and home-based BP measurements, are recommended by the ISH whenever possible. When used along with office BP measurements, they can detect white coat hypertension (WCH) and masked hypertension (MH). WCH is described as the event wherein untreated hypertension patients have high office BP readings but normal BP levels outside the medical setting. Meanwhile in MH, untreated hypertension patients have normal office BP but elevated ABPM readings. Both these conditions are detrimental to the patient, as WCH and MH may cause overtreatment and undertreatment, respectively [15].

The ISH guideline especially warrants out-of-office measurement for patients with high-normal BP to grade 1 hypertension, if feasible. This recommendation is also shared by the 2019 National Institute for Health and Clinical Excellence (NICE) and the 2018 Society of Cardiology/European Society of Hypertension guidelines [8,16].

The WHO guideline neither discusses out-of-office BP measurements nor explains WCH and MH. Instead, it mentions that along with other additional tests (such as lipid panel, glucose, and electrocardiogram), ABPM may pose as an economic barrier in the initiation of pharmacological treatment in less-resourced settings. The WHO guideline also only suggests a conditional recommendation for home-based self-care. While home-based BP monitoring may enhance BP control, there exists a research gap on its feasibility, costs, and effectiveness.

#### 2.1.4. Assessment of comorbidities and cardiovascular risk factors

Comorbidities, which are common in patients with hypertension, can affect CV risk and alter both non-pharmacological and pharmacological treatment plans. Some of the most commonly addressed comorbidities in hypertension guidelines are CVD, chronic kidney disease (CKD), and diabetes [17]. Testing enables the identification of comorbidities, secondary hypertension, and hypertension-mediated organ damage (HMOD). It facilitates cardiac risk classification and aids in better selection of medications [1]. Laboratory tests recommended by the WHO and ISH are adequate in screening for most comorbidities and secondary hypertension. However, the ISH guidelines advocate the use of meticulous investigations such as echocardiography and the aldosterone-renin ratio, which are costly and time-consuming. By contrast, the WHO guidelines favour more conventional tests, such as electrocardiogram, and prompt treatment.

It is estimated that more than half of the patients with hypertension have other CV risk factors. The most prevalent risk factors are metabolic syndrome, overweight/obesity, and lipid disorders, among others [2]. CV risk is an important consideration in initiating treatment. Predictably, a meta-analysis showed that those with the “highest risk” benefitted the most from BP-lowering treatment [18]. The ISH supports the assessment of risk factors in all hypertensive patients using a simple scoring system. The scoring system comprises BP measurement, HMOD, grade 3 CKD, diabetes, CVD, age, sex, and other factors. By contrast, the WHO suggests that patients with grade 1 hypertension do not require a CVD risk assessment before initiation of pharmacological treatment. The WHO considers CVD risk assessment as the most important parameter in patients with normal-high BP, perhaps to avoid overtreatment.

#### 2.1.5. Pharmacological therapy preferences

Regarding therapy, both the WHO and ISH favour combination therapy, preferably in the form of single-pill combinations (SPCs), which integrate two or more antihypertensive drugs into a single pill. SPC therapy has been cited to improve adherence and consistency and is associated with a reduction in mortality risk. The use of SPCs also prevents therapeutic inertia, which is a major contributor to the low rate of BP control worldwide [19,20]. Some disadvantages of SPC therapy include high cost, lack of flexibility, lack of evidence in randomised controlled trials, and bias from research authors and sponsors [21]. In terms of diuretic preference, the ISH favours thiazide-like diuretics compared with thiazide diuretics, whereas the WHO guidelines do not specify a preference. Previous analyses have concluded that thiazide-like diuretics are superior because of their greater efficacy and fewer side effects [22,23].

Both guidelines prefer a combination of two drug classes, such as angiotensin-converting enzyme inhibitors (ACEis) or angiotensin II receptor blockers (ARBs) combined with dihydropyridine calcium channel blockers (DHP-CCBs). In addition, the WHO considers thiazide/thiazide-like diuretics as potential choices in SPCs (Table 2). The ISH includes thiazide diuretics as a core drug in the treatment strategy, but only in the second step or after failure of the first step using a maximum combination dose. This issue was raised by Pareek *et al.* in their correspondence regarding the guidelines. They opined that the preference of DHP-CCBs over thiazide-like diuretics was not adequately supported by evidence [24]. For example, the ASCOT-BPLA trial showed that patients receiving combinations of DHP-CCB with ACEis had lower results in primary endpoints (fatal and non-fatal coronary heart diseases) compared with the combination of beta-blockers and thiazide diuretics. However, the result was not significant [429 vs 474; unadjusted HR = 0.90, 95% CI (0.79–1.02); *p* = .1502] [24,25]. Coincidentally, a similar result was found in the ALLHAT trial that favoured DHP-CCBs over chlorthalidone [11.3 vs. 11.5%; RR = 0.98, 95% CI (0.90–1.07)] [24,26]. Another issue raised by Pareek *et al.* was that the two included trials (ASCOT-BPLA and ACCOMPLISH) used weaker thiazide diuretics for comparison, which might have affected the results. Finally, they were critical of the ISH for not referring to the STOP-2 trial in their guidelines.

**Table 2.** Comparison of the pharmacological approaches between WHO and ISH guidelines.

Differences		
Characteristics	World Health Organisation (WHO)	International Society for Hypertension (ISH)
Single-pill combination therapy composition	Combination of ACEi/ARB or DHP-CCB or Thiazide/Thiazide-like diuretics	ACEi/ARB + DHP-CCB
Monotherapy consideration	Not mentioned	Low-risk grade 1 hypertension or in very old (≥80 years) patients
Resistant hypertension treatment strategy*	Refer to specialist	Adds spironolactone (12.5–50 mg once a day) or amiloride, doxazosin, clonidine, beta-blockers, etc. if spironolactone is contraindicated/unavailable
**Similarities**		
	Considers beta-blockers in ischaemic heart disease or post myocardial infarction	Considers beta-blockers at any treatment step if specific indications are present, e.g. heart failure, ischaemic heart disease, atrial fibrillation
	Considers CCB in heart failure, chronic kidney disease, and diabetes mellitus	
	Recommends triple-drug combinations when target BP is not achieved, despite having consumed dual drugs at maximum dose	
	Suggests utilisation of CCB in black patients	

ACEi: angiotensin-converting enzyme inhibitor; ARB: angiotensin II receptor blocker; BP: blood pressure; CCB: calcium channel blocker; DHP-CCB: dihydropyridine calcium channel blocker; ISH: International Society for Hypertension; WHO: World Health Organisation.

*seated office BP >140/90 mmHg in patients treated with ≥3 antihypertensives at optimal doses after excluding pseudoresistance.

Interestingly, Poulter *et al.* replied to this comment on behalf of the 2020 ISH Guidelines Committee insisting on the use of A + C (ACEis and DHP-CCBs) as first-line therapy for most patients. In response to the first issue of the lack of significance in the ASCOT-BPLA trial results, they defended their recommendations on the grounds that the trial was prematurely stopped as recommended by the data safety monitoring board because of serious adverse events. The trial also showed a significant difference in the proportion of patients who withdrew from the trial because of serious adverse events between the DHP-CCB-ACEi and beta-blocker-thiazide group [2 vs. 3%, *p* < .0001] [25,27]. Additionally, the authors disagreed with the rejection of the ACCOMPLISH results being only based on the drug choice. They also explained that the STOP-2 trial was not included because the study design did not compare A + C versus any other drug combination. The trial also only included patients aged 70–84 years, which is unrepresentative of the general population. Moreover, the trial showed no difference in cardiovascular outcomes between the CCB/ACEi and diuretics/beta-blocker regimen [27].

#### 2.1.6. Pharmacological intervention in resistant hypertension

The WHO and ISH guidelines have different pharmacological approaches for the treatment of resistant hypertension. The WHO recommends that physicians refer a case to specialists if BP control is not attained after the addition of fully adjusted doses of an ARB and thiazide/thiazide-like diuretics. While the ISH also suggests managing resistant hypertension by competent specialists with the resources to diagnose and treat such conditions, it provides an alternative of adding a fourth-line agent if a triple combination of drugs fails to control BP. The ISH advises an additional administration of low-dose spironolactone if the serum potassium is < 4.5 mmol/L and the estimated glomerular filtration rate is > 45 mL/min/1.73 m^2^. This recommendation is based on the PATHWAY-2 trial, which determined that resistant hypertension is mostly caused by salt retention and that mineralocorticoid receptor blockade by spironolactone can overcome this condition most effectively [28,29]. If spironolactone is unavailable, contraindicated, or not tolerated, amiloride, doxazosin, clonidine, eplerenone, beta-blockers, or any available class of antihypertensive that is not already in use should be administered. Additionally, a meta-analysis reiterated the superiority of spironolactone as a fourth-line agent in resistant hypertension [29,30].

#### 2.1.7. Views on beta-blockers

The WHO and ISH guidelines are in accord regarding their views on beta-blockers. Both consider the use of beta-blockers in presence of cardiac conditions, such as ischaemic heart disease or heart failure. In addition, both guidelines recommend the use of CCBs in patients with comorbidities such as diabetes mellitus, CKD, and heart failure. Both guidelines approve the administration of triple-drug combinations in patients consuming two drugs at a maximum dose if the target BP is still unattained.

#### 2.1.8. Hypertension in patients of African descent

Those of African descent are particularly prone to develop hypertension and HMOD at a younger age than those of other ethnicities. This vulnerability is attributed to physiological renal and CV differences [31–33]. In line with the results from various trials and reviews [34,35], both guidelines agree that black patients would particularly benefit from the administration of CCBs.

#### 2.1.9. Bp target and reassessment

Both the WHO and ISH recommend a target BP of <140/90 mmHg in patients without comorbidities. The ISH further defines optimal BP targets for those aged <65 and ≥65 years. The ISH aims for BP control within 3 months of pharmacological initiation, while the WHO does not define a target duration for achieving BP control. For patients with comorbidities and high CV risk, both guidelines suggest a target SBP of <130 mmHg. Caution should be exercised not to lower BP excessively. The ONTARGET and TRANSCEND trials demonstrated that lowering BPs below 120 mmHg demonstrated more harm than benefit, even in the elderly and those at high CV risk [36].

Another difference between these guidelines is the office BP targets. The WHO sets targets based on comorbidities (<140/90 mmHg in all hypertensive patients without comorbidities and <130 mmHg in hypertensive patients with known CVD or high-risk populations). This guideline is supported by systematic reviews [37,38] and clinical trials [39–41] that similarly concluded that individuals aged ≥ 65 years need a lower BP target. By contrast, the ISH uses a fixed number as the essential target (reduction by at least 20/10 mmHg or ideally to <140/90 mmHg); however, the optimal target is determined for specific age cohorts (121–129/71–79 mmHg for <65 years; <140/90 mmHg for ≥65 years). A meta-analysis of 61 prospective studies, comprising 1 million adults, revealed that each BP reduction by 20/10 mmHg correlated with a two-fold lower stroke death rate [42].

The WHO advises monthly reassessment of BP after starting BP-lowering therapy, with follow-up every 3–6 months after BP is controlled. Routine monthly follow-up post-therapy initiation was associated with important benefits shown in two clinical trials [39,40]. However, the difference between 3-month and 6-month follow-up intervals (after BP target was reached) was insignificant [43]. Meanwhile, the ISH does not provide recommendations on the follow-up frequency, although it does provide a 3-month deadline for BP control.

### 2.2. Authors’ observations

General comparisons between the WHO and the ISH guidelines are summarised in Table 3. First, both guidelines have implications for LMICs. The WHO hypertension guideline is the first global guideline to specify its use for LMICs, further dividing the recommendations according to the four levels of certainty (“very low,” “low,” “moderate,” and “high”). Meanwhile, the ISH divides its recommendation standards into “essential” and “optimal.” “Essential” recommendations are minimum standards of care that should be applied in both LMICs and HICs, whereas “optimal” means that the recommendation should be used whenever available [1,2].

**Table 3.** Comparison between the WHO and ISH guidelines.

World Health Organisation (WHO)	International Society for Hypertension (ISH)
**Similarities**	
LMIC-oriented recommendations	Includes recommendations for LMICs and HICs
Provides detailed evidence and rationale for each recommendation along with evidence-to-decision considerations	Presents a short paragraph containing evidence and rationale before each recommendation
**Differences**	
Breaks down recommendations into four levels of certainty (very low, low, moderate, and high)	Splits recommendation standards as “essential” and “optimal” based on current standards of care
Determines blood pressure targets based on known cardiovascular disease and patient’s risk	Determines blood pressure target based on patient’s age
Suggests nonphysician professionals to provide pharmacological therapy under some requirements	Discusses specific phenomena more commonly encountered in HICs such as white coat hypertension and masked hypertension
Discusses hypertension management in disasters and humanitarian crises	Pays more attention to ethnic and racial differences
Includes a section for hypertension in the context of Coronavirus disease	

LMICs: low- and middle-income countries; HICs: high-income countries; ISH: International Society for Hypertension; WHO: World Health Organisation.

The age-standardized prevalence of hypertension in LMICs increased by 7.7% from 2000 to 2010 [44]. Thus, guidelines are crucial for managing the real burden of hypertension in LMICs. Moreover, guidelines directly adopted from HICs cannot solve the problem of hypertension in LMICs. Additionally, these countries have several other problems related to the disease, such as low control and treatment rates, limited resources, and higher complication rates [2,7,44].

Various factors such as motives, authors’ backgrounds, methods, and the timing of writing are plausible explanations for the differences between the WHO and ISH guidelines. The WHO guidelines seem to encourage more aggressive treatment of hypertension, treating it as soon as possible while potentially sacrificing precision if it results in treatment delay. This rationale can be understood if the authors focussed on countries with higher prevalence of hypertension (mostly LMICs). The WHO also includes special recommendations for hypertension in the context of COVID-19 and humanitarian crises, which highly add to its relevance in current times.

Meanwhile, the ISH guidelines appear to prefer a more tailored and precise management. Although individualised to cater to each patient’s condition, this rigorous approach is probably more expensive and requires more time. Additionally, specialist services may not be available in all areas, especially in LMICs, and might not be covered by national health insurance plans [45]. This guideline acknowledges that some recommendations might not be feasible in low-resource settings and addresses them by classifying the recommendations into two categories.

To expand the usefulness of both guidelines in LMICs, the authors would like to address the possibility of their application in the “HEARTS” program created by the WHO in 2018 [46]. The program was mainly designed for limited-resource settings, which are present not only in LMICs, but also in other parts of the world. Furthermore, it also offers a framework to expand the extent of hypertension treatment in primary care setting and thus increases treatment accessibility for patients.

The program consists of six modules based on the abbreviation “HEARTS”: Healthy lifestyle, Evidence-based treatment protocols, Access to essential medicines and technology, Risk-based CVD management, Team-based care, and Systems for monitoring. In evidence-based treatment protocols, the WHO has provided some useful protocols with specific algorithms that include a selection of first-line treatments, including diuretics, CCBs, ACEi/ARBs, ACEi/ARBs + CCBs, CCBs + diuretics, and ACEi/ARBs + diuretics [47,48]. However, we recommend an update to the HEARTS program according to the latest hypertension guidelines available (as discussed in this review) to maintain its “evidence-based” status and continue combating the high burden of hypertension in LMICs.

This review has certain limitations, firstly, we did not compare the WHO or ISH recommendations with regional or national guidelines. Furthermore, we did not investigate the differences between WHO and ISH recommendations with population-specific guidelines (such as the Kidney Disease: Improving Global Outcomes guidelines, which supplied recommendations for hypertension in CKD). Lastly, we acknowledge the possibility of judgement bias in our observations since all the authors are from a lower-middle income country.

## 3. Conclusions

The WHO and ISH guidelines are both evidence-based and expert recommendations. Utilised in the correct settings, both guidelines have the potential to globally improve the control of hypertension. Local health departments should not hesitate to compare guidelines and modify, combine, and/or augment certain recommendations to formulate the best-suited evidence-based guidelines for their respective circumstances.

## Data Availability

The authors confirm that the data supporting the findings of this study are available within the article, reference number 1 and 2.
